# Supplementary material for: A More Diverse Cervical Microbiome Associates with Better Clinical Outcomes in Patients with Endometriosis: A Pilot Study
Source: Biomedicines. 2022 Jan 14;10(1):174. doi: 10.3390/biomedicines10010174 (PMC8774211; doi:10.3390/biomedicines10010174)
Supplement: Supplementary file 1 [file biomedicines-10-00174-s001.zip › biomedicines-1550821-supplementary.pdf]

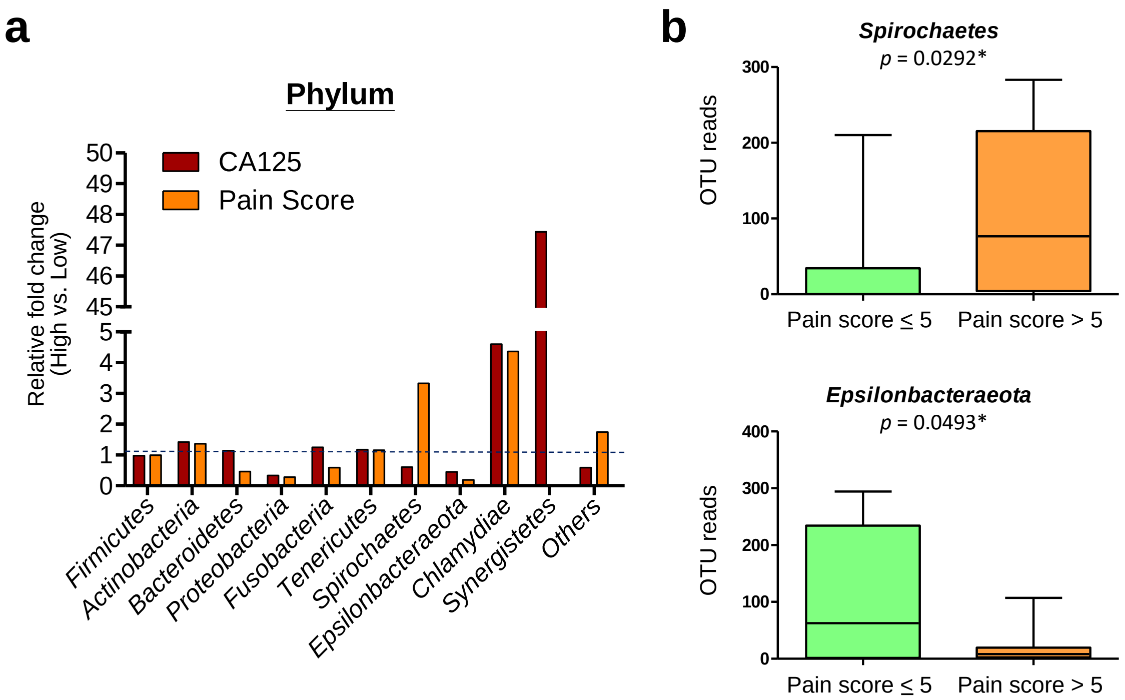

Figure S1. Relative abundance of the top-10 major phyla of cervical microbiomes in endometriosis patients with different CA125 levels and pain scores. (a) The abundance ratios of the top-10 major phyla between patients with high (> 35 U/mL) and low CA125 (≤ 35) levels (red) or high (> 5) and low (≤ 5) pain scores (orange). (b) Two-group comparison by t-test analysis defines the statistically significant changes of the top-10 major phyla between patient groups.

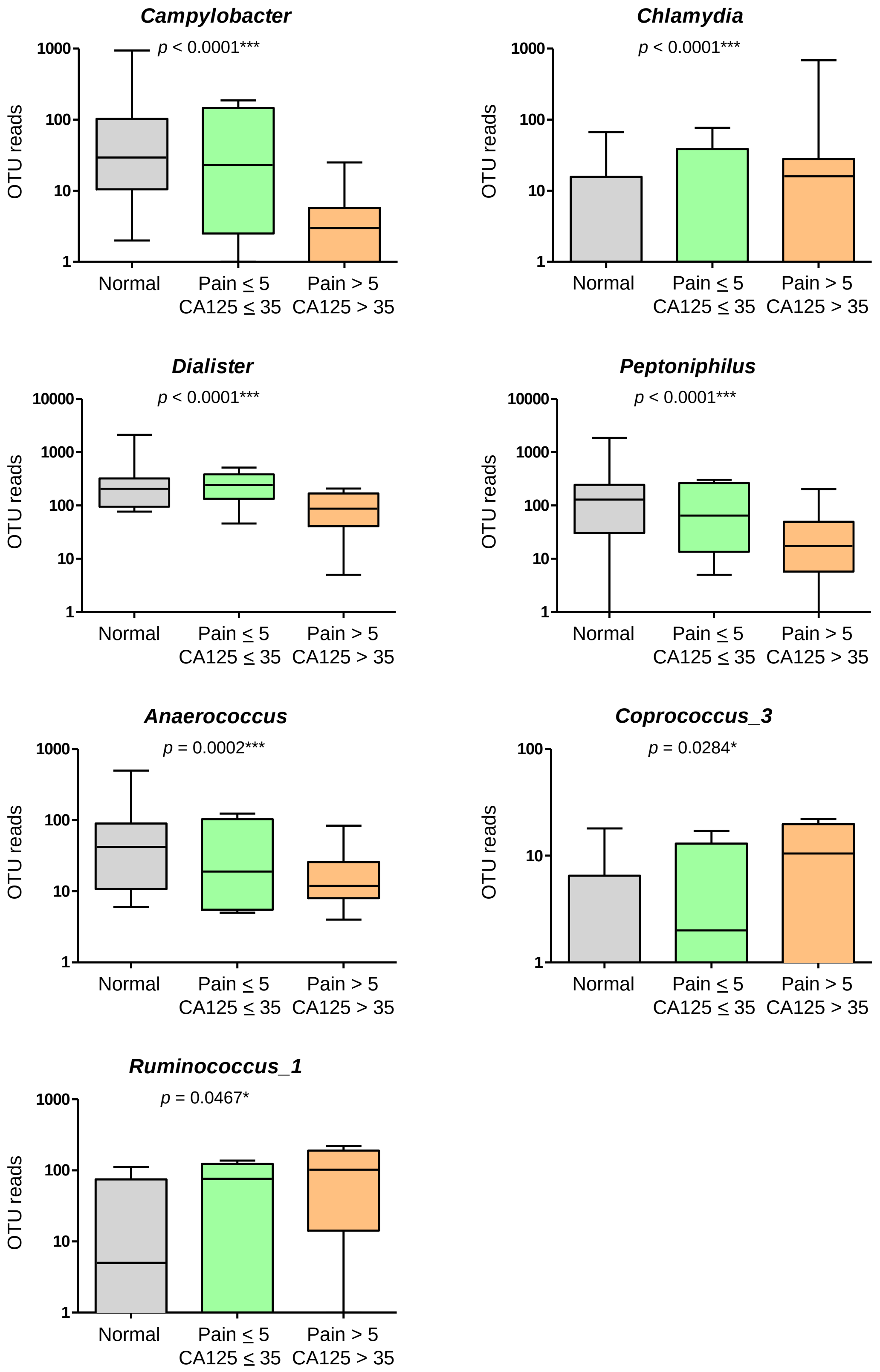

Figure S2. One-way ANOVA analysis defines key altered genera of cervical microbiome that show consistent up- or down-regulation among normal control, double-negative and double-positive patients with endometriosis.

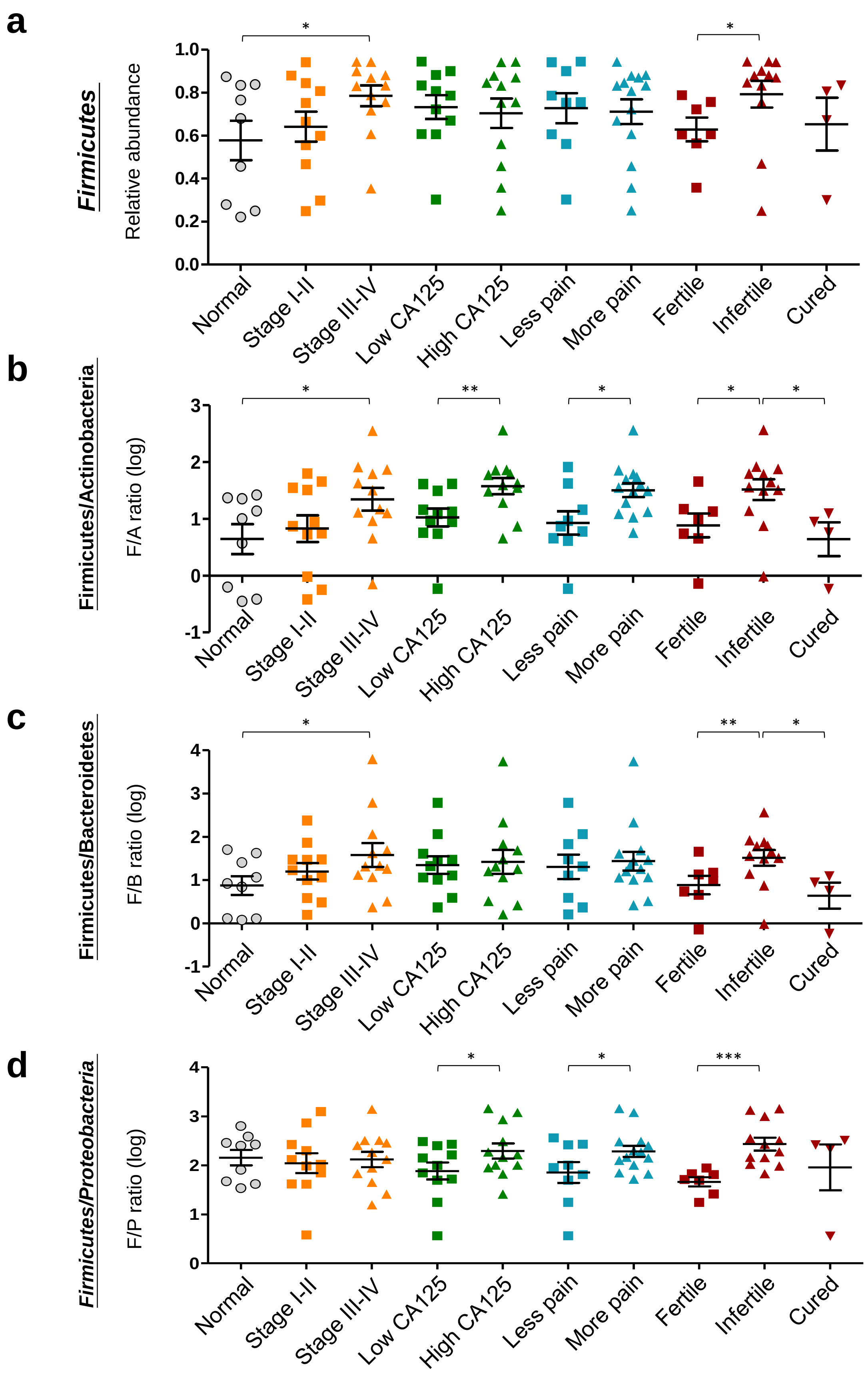
a
Firmicutes
Relative abundance
1.0
0.8
0.6
0.4
0.2
0.0
*
*
Normal
Stage I-II
Stage III-IV
Low CA125
High CA125
Less pain
More pain
Fertile
Infertile
Cured
b
Firmicutes/Actinobacteria
F/A ratio (log)
3
2
1
0
-1
*
**
*
*
*
c
Firmicutes/Bacteroidetes
F/B ratio (log)
4
3
2
1
0
-1
*
**
*
d
Firmicutes/Proteobacteria
F/P ratio (log)
4
3
2
1
0
*
*
***

Figure S3. Species abundance and their ratios in endometriosis patients with different clinical features. (a) The abundances of *Firmicutes* and the ratios of (b) *Firmicutes*/*Actinobacteria* (F/A), (c) *Firmicutes*/*Bacteroidetes* (F/B), (d) *Firmicutes*/*Proteobacteria* (F/P) were compared between patients with different clinical features. Two-group comparison was performed by t-test, and three-group comparison was done by one-way ANOVA.

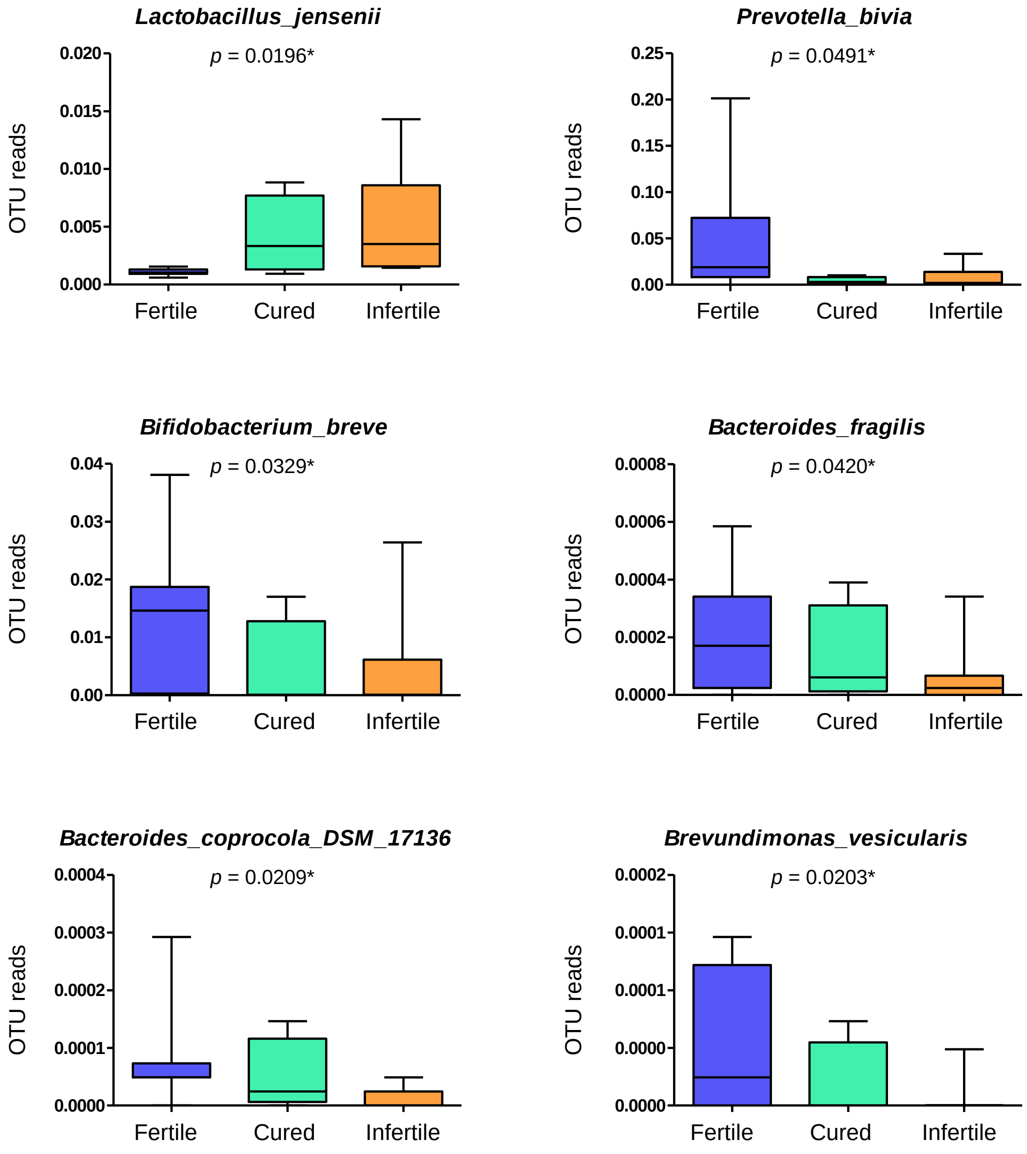

Figure S4. One-way ANOVA analysis defines key altered species of cervical microbiome that show consistent up- or down-regulation among fertile, cured and infertile patients with endometriosis.

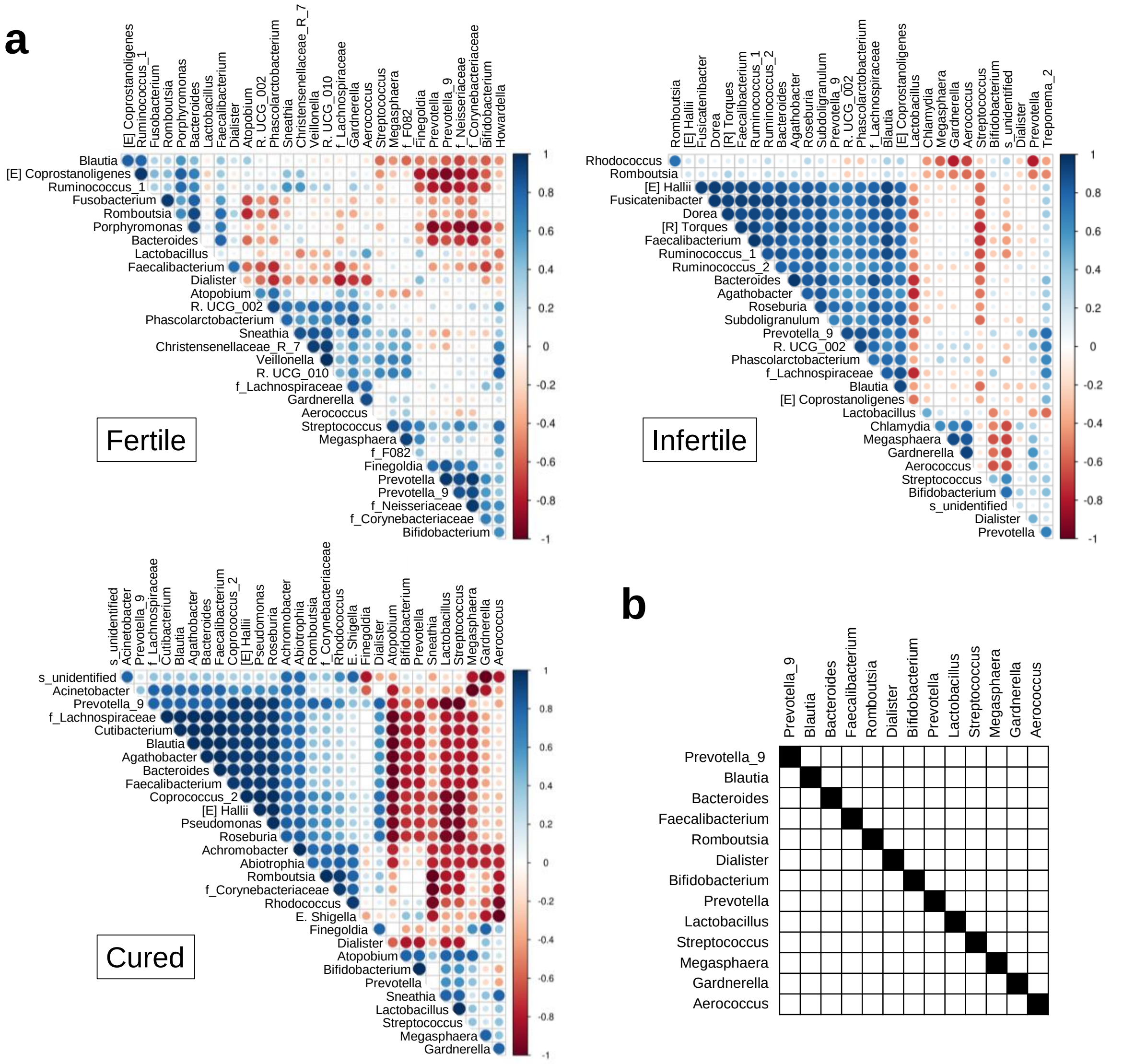

Figure S5. Ecological relationships among cervical microbes in patients with different reproductive ability. (a) Spearman correlation matrices reveal the significant inter-genus long-term drifts of cervical microbiomes from patients with different reproductive ability. The circle size indicates the absolute value of a correlation coefficient and the color indicates the positive (blue) or negative (red) correlation. (b) The common Spearman correlation matrix shared by different patient groups.

Table S1. Summary of clinicopathological features of 23 patients with endometriosis

Patient No.	Age	AFS stage	CA125 (U/mL)	VAS pain	Fertility	ENZIAN Score								Cyst No.	Largest cyst size (cm)
						A	B	C	FA	FB	FU	FI	FO		
E1	39	IV	89.0	8	fertile	1	1	1	1	0	0	0	0	3	4.7
E2	34	I	46.8	10	infertile	1	2	1	1	1	0	0	0	1	4.0
E3	32	I	74.8	7	infertile	1	2	1	1	0	0	1	0	3	4.0
E4	35	IV	47.6	9	infertile	1	2	1	1	0	0	1	0	1	5.0
E5	39	IV	54.5	8	infertile	1	2	1	1	0	0	0	0	1	7.7
E6	43	IV	25.2	3	fertile	1	1	0	1	0	0	0	0	1	7.0
E7	54	IV	29.2	3	fertile	1	2	1	0	0	0	0	0	1	10.0
E8	35	IV	20.2	8	fertile	0	1	0	1	0	0	0	0	1	7.0
E9	37	III	29.0	6	cured	0	0	0	0	0	0	0	0	3	9.0
E10	29	IV	27.5	8	cured	1	1	1	0	0	0	0	0	1	8.0
E11	26	IV	67.6	3	fertile	1	2	0	1	0	0	0	0	3	4.0
E12	46	II	64.4	3	fertile	1	2	0	0	0	0	0	0	2	5.3
E13	29	III	63.4	8	infertile	1	2	1	1	0	0	0	0	1	8.0
E14	30	I	147.0	9	infertile	0	0	0	0	0	0	0	0	1	9.0
E15	24	IV	34.4	5	infertile	0	1	0	1	0	0	0	0	1	6.0
E16	38	IV	23.6	2	infertile	1	1	0	0	0	0	0	0	2	4.3
E17	35	II	19.1	3	cured	0	0	0	0	0	0	0	0	1	7.0
E18	38	IV	32.4	8	infertile	0	1	0	0	0	0	0	0	1	6.0
E19	36	I	22.4	8	fertile	0	1	0	0	0	0	0	0	1	3.0
E20	36	I	19.1	6	cured	0	0	0	0	0	0	0	0	1	8.0
E21	28	I	43.6	3	infertile	0	0	0	0	0	0	0	0	1	4.0
E22	33	II	42.1	2	infertile	0	0	0	0	0	0	0	0	2	4.5
E23	39	II	56.7	1	infertile	0	0	0	0	0	0	0	0	1	10.0
